# Validation and feasibility of a deep learning-based reconstruction technology in 5.0 tesla knee joint MR imaging

**DOI:** 10.3389/fradi.2026.1776035

**Published:** 2026-02-19

**Authors:** Pan Wang, Zhigang Li, Chuan Zhu, Ran Mu, Chang Liu, Jing Yang, Lixin Du

**Affiliations:** 1 Department of Radiology, Shenzhen Longhua District Central Hospital, Shenzhen, China; 2 United Imaging Healthcare, Shanghai, China

**Keywords:** 5.0 tesla MRI, CNN, deep learning reconstruction, knee joint, SNR

## Abstract

**Purpose:**

This study aimed to evaluate the feasibility of a deep learning-based reconstruction (DLR) algorithm for optimizing conventional 5.0 Tesla knee joint MR protocols.

**Methods:**

This prospective study enrolled 69 patients who underwent both knee arthroscopy and 5.0 Tesla knee joint MR examinations using the conventional protocols before and after a DLR process with different levels. The DLR technique was applied to original images to denoise and improve their quality. Two radiologists independently measured the signal-to-noise ratio (SNRs) in cartilage, meniscus, bone, ligament, and muscle, and graded image quality from the dimensions of different tissues' delineation clarity, global artifact severity, and overall image quality using a 5-point Likert scale. Moreover, the diagnostic performance was evaluated with different types of images, compared to the results of knee arthroscopy. Cohen's kappa test was employed to assess the agreement of image quality scoring and diagnosis.

**Results:**

Compared to conventional images, those DLR ones demonstrated significant improvement in SNRs, with the increasement of 12.61% to 350.63% across various sequences. Two radiologists showed good-to-excellent agreement in image quality assessment, with kappa values ranging from 0.72 to 0.82. Regarding diagnostic performance, the DLR images moderately outperformed the non-DLR ones, as evidenced by a bit higher diagnostic agreement with the results of knee arthroscopy (DLR: kappa = 0.908–1; non-DLR: kappa = 0.882–0.963).

**Conclusions:**

The DLR technique could improve 5.0 Tesla knee MR images' quality and obtain as least equal diagnostic efficiency without extra scan time, demonstrating its potential clinical applicability.

## 1 Introduction

The knee joint, a primary weight-bearing structure, is particularly susceptible to traumatic injuries and degenerative pathologies (1). The increasing incidence of sports-related knee injuries has been associated with rising living standards and greater participation in physical activities (2). Magnetic resonance imaging (MRI) serves as a cornerstone in knee pathology diagnosis owing to its non-invasive nature, superior spatial resolution, and excellent soft-tissue contrast (3, 4). High-resolution 2D fast spin-echo (FSE) and proton density-weighted (PD) sequences are critical for delineating intricate anatomical structures and discriminating injury subtypes (5). Prolonged acquisition durations elevate motion artifact susceptibility, while k-space-related noise and Gibbs artifacts compromise diagnostic image quality (6). These limitations amplify in high-field systems (e.g., 5.0T), wherein theoretical resolution advantages are counterbalanced by compromised noise mitigation and acquisition efficiency (7).

To address these challenges, contemporary MRI acceleration techniques have adopted strategies such as parallel imaging (PI) and compressed sensing (CS). In parallel imaging approaches (e.g., SENSE/GRAPPA), the number of phase-encoding steps is reduced proportionally with the acceleration factor. This reduction leads to spatially varying noise amplification, which is particularly pronounced in central image regions where coil sensitivity profiles change most markedly. Consequently, background noise increases, the SNR declines, and residual aliasing artifacts emerge—with SNR reductions exceeding 60% at an acceleration factor of (8, 9). Alternatively, CS leverages image sparsity through L1-norm-constrained reconstruction to enable substantial k-space undersampling (10, 11). Despite its potential, CS suffers from inherent drawbacks: its nonlinear reconstruction tends to amplify high-frequency noise and introduce structured artifacts such as Gibbs ringing, which can adversely affect diagnostic accuracy.

Emerging artificial intelligence (AI) advancements, particularly DLR methods, have transformed medical imaging paradigms. While DLR has been validated in musculoskeletal and prostate imaging (12–15), its translational potential in 5.0T knee MRI remains uninvestigated. This study was to evaluate the DLR's capability for optimizing image quality and workflow efficiency in 5.0T knee MRI, addressing precision diagnostic requirements in high-field imaging systems.

## 2 Materials and methods

The study protocol received Institutional Review Board approval with written informed consent secured from all participants.

### 2.1 Patients and MR protocols

**Figure 1:**
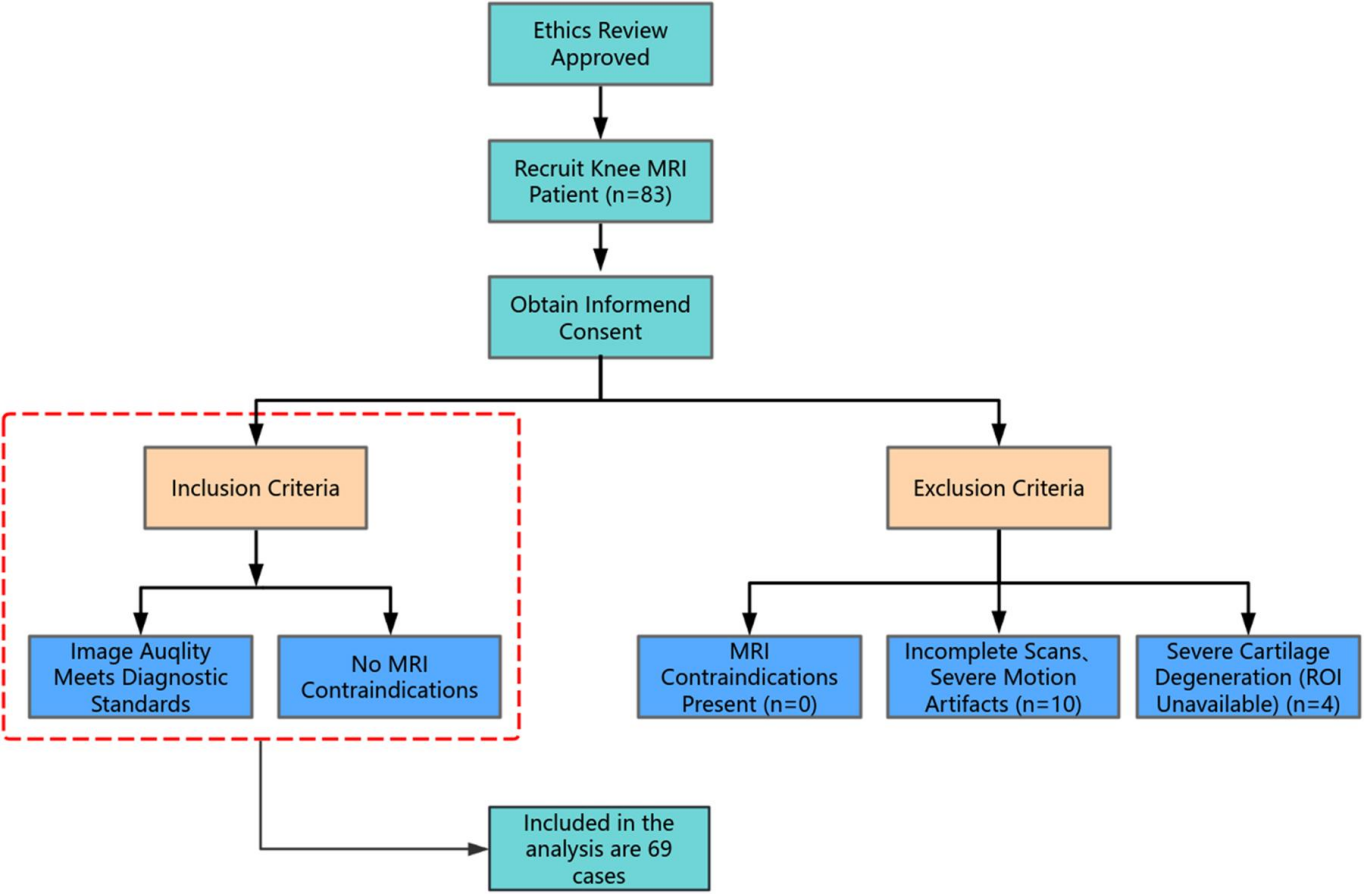
Patient screening workflow.

This study enrolled a prospective cohort of 83 patients scheduled to undergo knee MRI examinations between June and July 2025. Following screening, 69 patients were ultimately included.The knee arthroscopy-MRI interval was limited to 10 days across all patients. The cohort included 38 males and 31 females, aged 13–61 years (mean age: 36.63 ± 11.82). All patients underwent 5.0T knee MRI on a clinical scanner (uMR Jupiter, United Imaging Healthcare, China). The acquired data were processed using a deep learning reconstruction (DLR) algorithm at three distinct intensity levels: weak, medium, and strong. Inclusion Criteria: ①Clinical diagnosis or suspected knee pathologies warranting MRI evaluation. ②Images fulfilled diagnostic quality criteria. Exclusion Criteria: ①Contraindications to MRI (e.g., cardiac pacemakers, metallic implants)(*n* = 0). ②Insufficient imaging sequences or absence of raw reconstruction data (*n* = 1). ③Severe cartilage degeneration precluded region-of-interest (ROI) sampling (*n* = 4). ④Positional shifts during scanning (*n* = 4). ⑤Severe motion artifacts (*n* = 5). Figure 1 illustrates the patient screening workflow.

MRI was performed using a 5.0T scanner equipped with a 24-channel knee coil. During the examination, all patients were positioned supine in a feet-first orientation. Sequences included T2-weighted fast spin-echo (T2-FSE), T1-weighted FSE (T1-FSE), and density-weighted (PD)-weighted acquisitions. Parameters are summarized in Table 1.

**Table 1:** Detailed parameters and scanning time for knee joint 2D MRI sequences.

Sequence	TR (ms)	TE (ms)	FOV (mm)	Slice thickness(mm)	Slice gap (%)	NEX	Intralayer resolution (mm)	Scanning time (s)
Sagittal T2WI FSE	4,203	70.09	140 × 140	3	10	1.7	0.24 × 0.20	93
Sagittal PD FSE FS	2,622	30.10	140 × 140	3	10	1.7	0.33 × 0.42	105
Sagittal T1WI FSE	600	6.70	140 × 140	3	10	1.6	0.21 × 0.17	99
Coronal PD FSE FS	2,521	24.35	140 × 140	3	10	1.6	0.25 × 0.21	111

PD, proton density; TR, repetition time; TE, echo time; NEX, number of excitations.

### 2.2 The DLR postprocess

DLR resolves the longstanding MRI trilemma (scan time, SNR, spatial resolution) through adaptive neural network-driven optimization (16). Traditional approaches necessitate trade-offs between scan duration and resolution to optimize SNR, k-space noise refers to the inherent random signal fluctuations in the raw acquisition data, which manifest as a granular texture in the background or uniform tissue regions of the reconstructed image. Gibbs artifacts are oscillatory stripes of alternating brightness and darkness that appear along sharp tissue boundaries in the image, resulting from the truncation of high-frequency data in k-space, while k-space-related noise and Gibbs artifacts persist with conventional mathematical frameworks (17, 18). The DLR, named DeepRecon (United Imaging Healthcare, China), implements lightweight-optimized convolutional neural networks (CNNs) to mitigate noise and improve spatial resolution while preserving acquisition durations. The CNN model used in this study is an end-to-end reconstruction framework. The input consists of undersampled raw k-space data, and the output is the corresponding fully-sampled image. The network employs a dual-domain learning architecture that simultaneously optimizes image-domain fidelity and k-space data consistency. An embedded data consistency module ensures the reconstruction conforms to the actual physical measurements. The model is trained via supervised learning to directly learn a nonlinear mapping from accelerated acquisitions to high-quality images. Rather than relying on fixed mathematical priors, the CNN learns a direct, non-linear mapping from undersampled k-space to high-quality images. Its key advantage lies in adaptive, context-aware processing—simultaneously suppressing noise and mitigating artifacts such as Gibbs ringing—while preserving true anatomical edges through dual-domain consistency constraints. The novelty resides in dual-domain (image/k-space) fidelity constraints: Image domain, harnessing multicenter training data encompassing anatomical and contrast variations, DLR enables discriminative noise-signal separation while preserving anatomical fidelity (19, 20); k-space domain, the fidelity module enforces data consistency between reconstructed images and raw k-space data through loss function constraints. Iterative optimization and matrix fusion synergistically improve accuracy. Experimental results demonstrate 44% SNR enhancement vs. conventional methods while maintaining image uniformity (13, 21–22).

The study comprised two phases: standard knee MRI acquisitions followed by DLR application with varying reconstruction intensities (weak/medium/strong), the Weak, Medium, and Strong reconstruction levels, which are integrated presets of the DLR algorithm balancing denoising and detail preservation. All images for different levels were generated from the same raw k-space data in an offline, parallel reconstruction process to ensure a controlled comparison.

#### 2.3.1 Quantitative assessment of image quality

Four conventional sequences (Sagittal T2WI FSE, Sagittal PD FSE FS, Sagittal T1WI FSE, Coronal PD FSE FS) were reconstructed at three DLR levels (weak/medium/strong), generating 12 reconstructed groups. Two radiologists (with 23 and 11 years of experience, respectively) manually segmented regions of interest (cartilage, meniscus, bone, ligament, muscle, and background) on sagittal and coronal planes using uOmnispace software. ROIs were standardized (1.5 mm^2^), with four ROIs/tissue averaged to calculate mean signal intensity. Background noise was quantified as the mean SD from four background ROIs. The formula for calculating SNR is given in Equation 1:

$$\mathrm{SN}R_{\mathrm{tissues}}=\frac{\mathrm{Signa}l_{\mathrm{tissues}}}{SD_{\mathrm{background}}}$$ (1)

The Signal_tissues_ represents the signal mean of the ROI for cartilage, meniscus, bone, ligament, and muscle. The SD_background_, is the average standard deviation of the ROI signal in the background area.

#### 2.3.2 Qualitative assessment of image quality

Two radiologists from distinct subspecialties performed blinded evaluations, independently assessing 16 image series/patient. During assessment, image metadata were blinded while patient images were randomized per reader. A 5-point scoring system assessed cartilage, meniscus, osseous structures, ligaments, and musculature in each patient. Assessment metrics comprised tissue delineation clarity, global artifact severity, and overall image quality, where higher scores corresponded to superior image quality. The image quality grading criteria are as follows: 5 (Excellent) indicated optimal diagnostic value with nearly artifact-free visualization, demonstrating exceptional structural clarity and delineation; 4 (Good) represented reliable diagnostic capability for most clinical scenarios despite minor artifacts that did not obscure structural evaluation; 3 (Fair) denoted acceptable diagnostic quality for routine interpretation with moderate artifacts partially limiting structural assessment; 2 (Limited) described severe localized artifacts/noise significantly compromising structural delineation, permitting only provisional diagnostic judgments; 1 (Non-diagnostic) reflected extensive artifacts/noise rendering anatomical features indiscernible and precluding meaningful clinical evaluation.

### 2.4 Diagnosis

Two radiologists with extensive diagnostic experience (25 years and 21 years respectively) independently diagnosed each patient's cartilage, meniscus, bone, ligaments, and muscles (in 2D sequences Sagittal T2WI FSE, Sagittal PD FSE FS, Sagittal T1WI FSE, Coronal PD FSE FS) using the following grading system. Grading system:

Meniscus (medial/lateral): Normal (0), degeneration (1), tear (2).

Cartilage (patellofemoral/tibiofemoral): Normal (0), degeneration (1), injury (2).

Ligaments (ACL/PCL): Normal (0), injury (1), tear (2).

Bone: Normal (0), bone marrow edema (1), cyst (2).

Muscle: Normal (0), edema (1), tear (2).

### 2.5 Statistical analysis

Statistical analysis was conducted with statistical software (SPSS, v. 27.0, R software). This study employed a combined quantitative and qualitative approach to evaluate the impact of deep learning reconstruction (DLR) on knee MRI image quality and diagnostic efficacy. Statistical analyses employed Wilcoxon signed-rank tests to assess tissue-specific SNR variations between reconstruction intensities (weak/medium/strong), given that all comparisons were performed on measurements from the same cohort of patients, paired statistical tests were mandatory. Comparisons between two related conditions were performed using the Wilcoxon signed-rank test. Pairwise comparisons were performed using Wilcoxon signed-rank tests α=0.05, specifically comparing the conventional reconstruction with each DLR level (Weak, Medium, Strong). Cohen's weighted kappa quantified interobserver agreement (two radiologists) and intra-observer concordance (radiologist vs. arthroscopic reference standard). The calculation formula is as follows: $\;\kappa =P_{o}-P_{e}/1-P_{e}$, where $P_{o}$ is the observed proportion of agreement (i.e., the relative observed agreement among readers), $P_{e}$ is the expected proportion of agreement by chance (23). Knee arthroscopy served as the diagnostic reference standard, with kappa analysis comparing DLR and non-DLR diagnostic accuracy. The kappa values were interpreted as: <0.20, poor; 0.21–0.40, fair; 0.41–0.60, moderate; 0.61–0.80, substantial; 0.81–1.00, near-perfect agreement. Statistical significance was defined as *p* < 0.05.

## 3 Results

### 3.1 Quantitative assessment results

**Figure 2:**
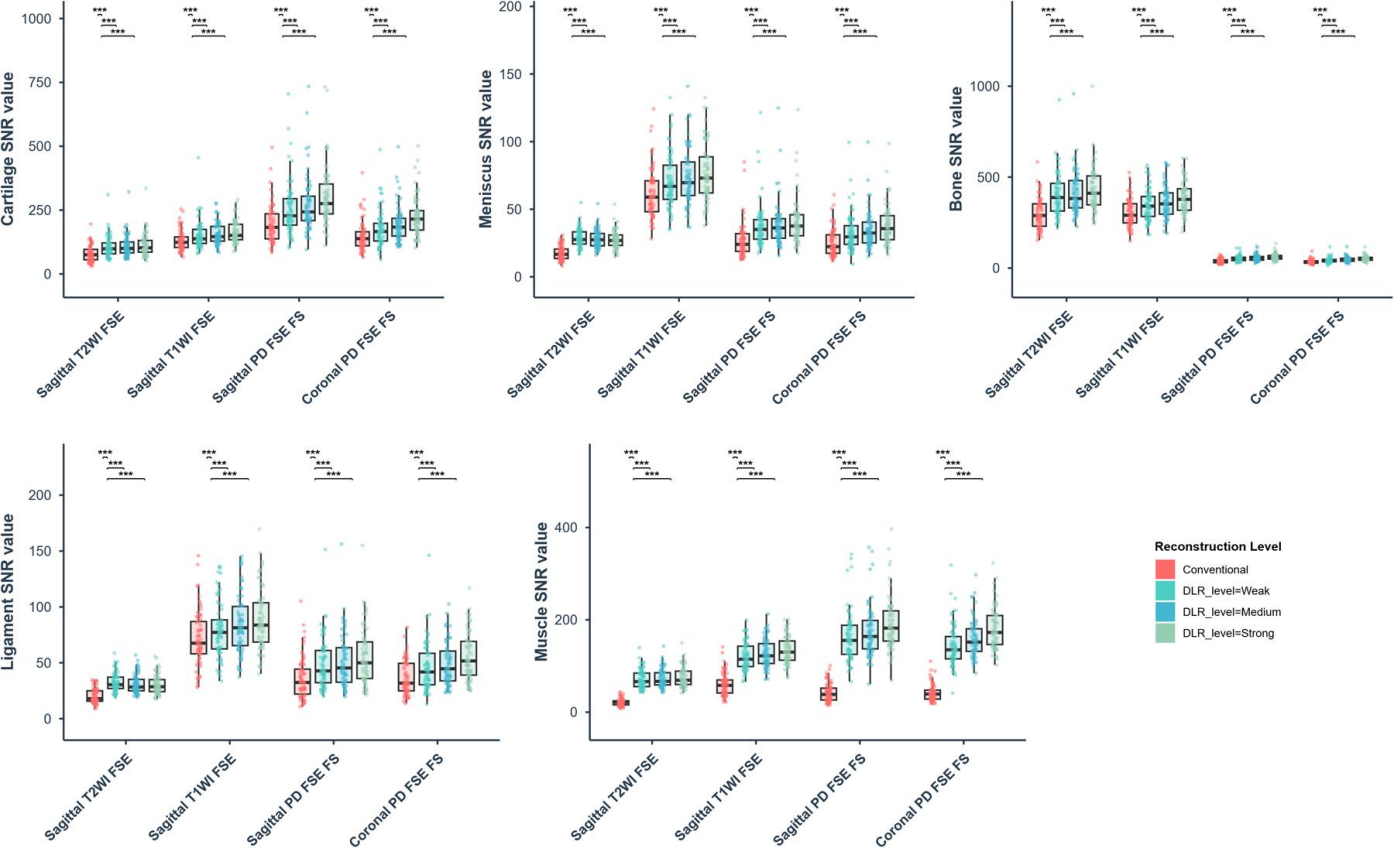
Box plots of signal-to-noise ratio (SNR) values for cartilage, meniscus, bone, ligament, and muscle across different reconstruction levels, where the conventional and various deep learning reconstruction (DLR) images are distinguished by color. (***P < .001).

Wilcoxon signed-rank tests assessed SNR differences between conventional and DLR images across intensity levels (weak/medium/strong). Compared to conventional images, the DLR ones demonstrated significant SNR improvements (12.61–350.63%) across all tissue types evaluated in this study (*p* < 0.001), as detailed in Table 2 and Figure 2. Tissue-specific SNR increases ranged: Cartilage 17.23–54.16%; Meniscus 13.73–63.27%; Osseous 13.47–54.8%; Ligament 12.61–56.95%; Musculature 100.31–350.63%, with the most pronounced improvement in musculature. Moreover, the DLR performance exhibited sequence dependency: T2WI and PDWI demonstrated enhanced DLR sensitivity vs. T1WI, with quantifiably superior SNR improvements.

**Table 2:** Quantitative comparison of DLR and non-DLR MRI for various tissues across different imaging sequences.

SNR	Conventional	Different levels of DLR		
		level = weak	level = medium	level = strong
Sagittal T2WI FSE				
Cartilage	78.50 ± 31.67	105.66 ± 41.89	108.56 ± 42.81	113.06 ± 44.42
Meniscus	17.51 ± 5.25	28.59 ± 6.82	27.72 ± 6.66	27.52 ± 6.64
Bone	301.53 ± 86.50	400.57 ± 115.98	411.91 ± 117.34	430.96 ± 121.06
Ligament	20.44 ± 6.80	32.08 ± 8.39	31.00 ± 8.39	30.46 ± 8.53
Muscle	21.32 ± 8.12	71.60 ± 20.01	72.90 ± 20.23	75.39 ± 21.24
Sagittal PD FSE FS				
Cartilage	194.72 ± 73.82	256.18 ± 104.57	272.38 ± 107.66	300.18 ± 111.56
Meniscus	26.65 ± 12.74	37.76 ± 16.55	38.31 ± 16.38	40.78 ± 17.04
Bone	38.85 ± 12.22	52.54 ± 16.30	55.59 ± 16.89	60.14 ± 17.22
Ligament	36.11 ± 18.71	49.20 ± 22.63	51.32 ± 23.76	55.20 ± 25.05
Muscle	42.41 ± 19.95	162.93 ± 54.72	173.40 ± 55.22	191.12 ± 57.38
Sagittal T1WI FSE				
Cartilage	129.34 ± 39.79	151.62 ± 54.50	155.50 ± 41.18	164.03 ± 42.26
Meniscus	61.68 ± 18.76	70.15 ± 19.91	73.32 ± 20.83	76.50 ± 21.33
Bone	305.16 ± 77.45	346.25 ± 85.98	364.73 ± 88.18	384.49 ± 89.81
Ligament	71.07 ± 24.58	80.03 ± 25.25	84.04 ± 26.21	88.20 ± 27.33
Muscle	60.15 ± 24.60	120.49 ± 29.65	127.29 ± 30.34	134.02 ± 30.29
Coronal PD FSE FS				
Cartilage	148.11 ± 57.57	177.80 ± 71.26	195.34 ± 70.40	224.09 ± 77.91
Meniscus	25.54 ± 10.12	32.66 ± 14.00	34.50 ± 13.60	37.88 ± 14.26
Bone	34.30 ± 11.73	42.33 ± 14.61	46.11 ± 14.04	51.90 ± 14.14
Ligament	36.45 ± 15.09	45.72 ± 21.72	48.97 ± 19.08	55.15 ± 21.35
Muscle	41.96 ± 18.57	143.38 ± 44.81	157.91 ± 41.67	180.26 ± 44.58

The SNR of each organization is presented in the form of “mean ± standard deviation”. All comparisons between conventional imaging and different DLR levels yielded statistically significant differences, with *p*-values < 0.001.

### 3.2 Qualitative assessment results

**Figure 3:**
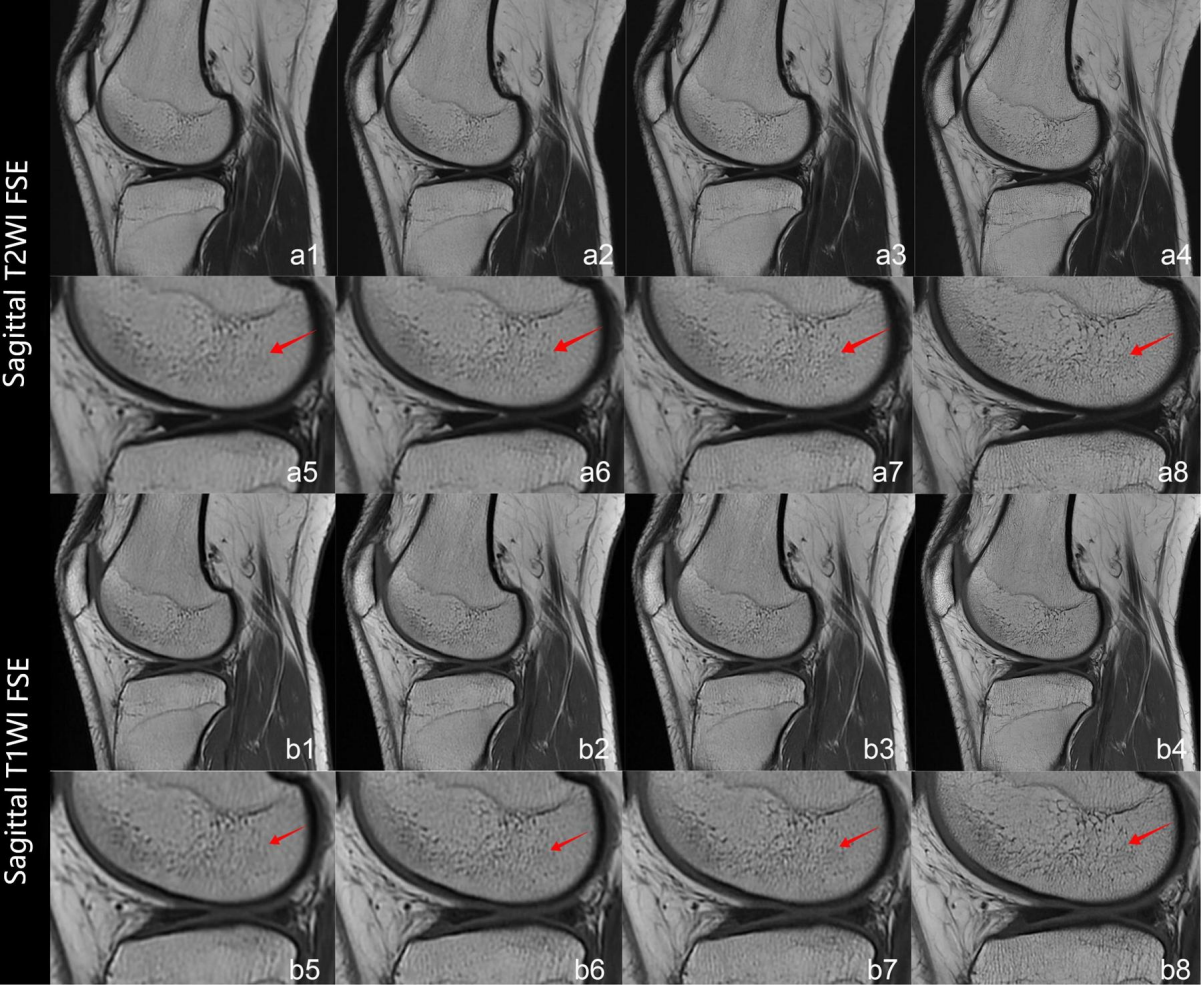
Panels **a1–a8** depict sagittal T2WI FSE sequences, while panels **b1–b8** represent sagittal T1WI FSE sequences. Conventional reconstructions (**a1,b1**) and DLR at weak/medium/strong intensities (**a2–a4,b2–b4**) are comparatively displayed. Magnified views of boxed regions (**a1–a4,b1–b4**) correspond to **a5–a8,b5–b8**. Red arrows demonstrate progressive trabecular bone visualization enhancement correlating with reconstruction intensity.

**Figure 4:**
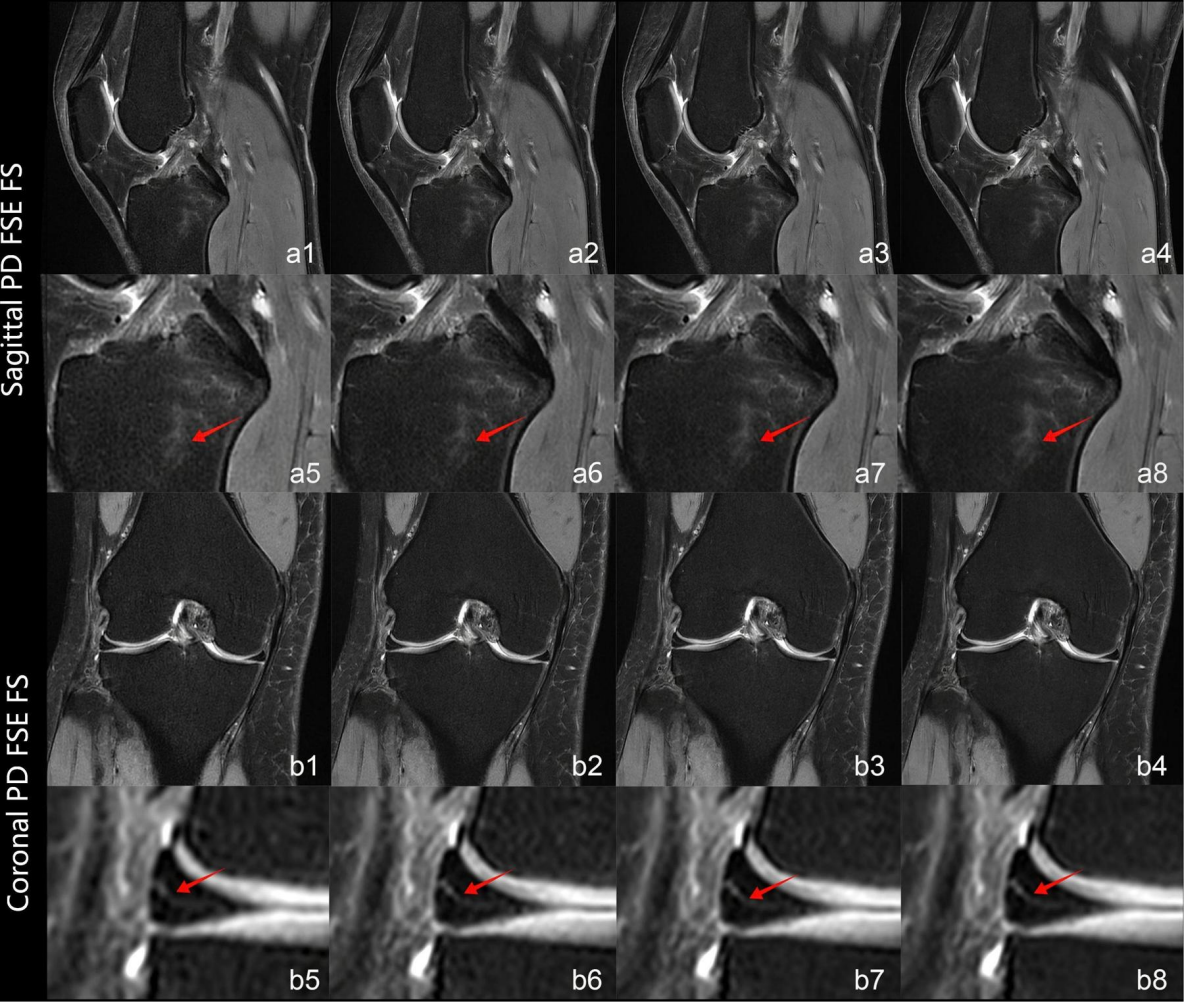
Panels **a1–a8** show sagittal PD FSE FS sequences, and panels **b1–b8** display coronal PD FSE FS sequences. Conventional reconstruction images are illustrated in **a1** and **b1**. Images reconstructed with DLR at levels weak, medium, and strong are shown in **a2–a4** and **b2–b4**. Magnified views of **a1–a4** and **b1–b4** are provided in **a5–a8** and **b5–b8**. In sagittal views (**a5–a8**), red arrows indicate gradual improvement in bone marrow edema delineation and noise reduction with higher reconstruction levels. Coronal magnified views (**b5–b8**) illustrate improved meniscal tear delineation correlating with increasing reconstruction intensities.

**Figure 5:**
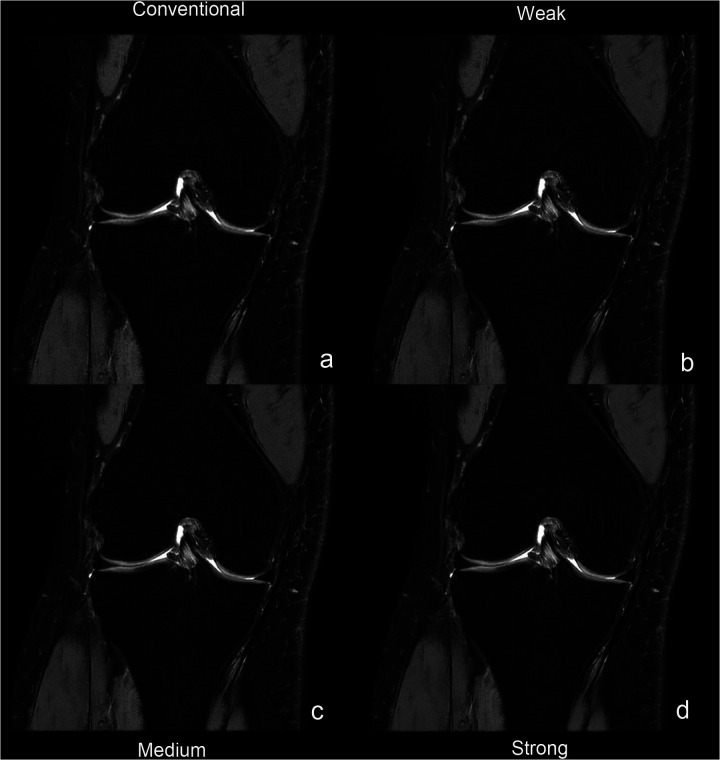
Panels **a–d** represent coronal PD FSE FS sequences: **(a)** conventional image; **(b–d)** DLR images at levels weak **(b)**, medium **(c)**, and strong **(d).**

Interobserver agreement was quantified between two radiologists using Cohen's weighted *κ* coefficient. Table 3 provides the complete results of the image quality analysis. The interreader agreement was substantial for all quality measures (*κ* = 0.72–0.82). The two readers consistently scored the overall image quality as good or very good [rating, ≥4 (IQR, 4–5 or higher)]. The artifacts with DLRs were minimal or nearly artifact-free [rating, ≥4 (IQR, 4–5 or higher)] and the artifacts without DLR was moderate [rating, 4 (IQR, 4–4)]. On images processed with DLR, all evaluated anatomical structures, including cartilage, meniscus, bone, ligament, and muscule—were good or very good [rating, ≥4 (IQR, 4–5 or higher)]. At the strong reconstruction intensity level, these structures consistently attained the maximum score of 5. By comparison, conventional methods yielded scores of 3 or above for all anatomical structures. Figure 3 illustrates progressively enhanced trabecular bone visualization correlating with reconstruction intensity (arrows), while Figure 4 demonstrates superior lesion margin definition. Figure 5 demonstrates progressive noise reduction in fat-suppressed sequences with increasing reconstruction intensities, validating image quality enhancements while preserving acquisition durations and diagnostic fidelity.

**Table 3:** Results of subjective scoring by two radiologists.

Score	Conventional	Different levels of DLR			kappa
		level = weak	level = medium	level = strong	
Cartilage	4 (3, 4–4, 5)	4 (4, 4–5, 5)	4 (4, 4–5, 5)	5 (4, 5–5, 5)	0.75 (0.70–0.80)
Meniscus	4 (4, 4–5, 5)	5 (4, 5–5, 5)	5 (4, 5–5, 5)	5 (4, 5–5, 5)	0.76 (0.71–0.81)
Bone	5 (4, 5–5, 5)	5 (4, 5–5, 5)	5 (4, 5–5, 5)	5 (4, 5–5, 5)	0.76 (0.72–0.81)
Ligament	3 (3, 4–4, 5)	4 (3, 4–4, 5)	4 (4, 4–5, 5)	4 (4, 4–5, 5)	0.72 (0.68–0.79)
Muscle	5 (4, 5–5, 5)	5 (4, 5–5, 5)	5 (4, 5–5, 5)	5 (4, 5–5, 5)	0.82 (0.78–0.87)
Artifact	3 (2, 4–4, 5)	4 (2, 4–4, 5)	5 (4, 5–5, 5)	5 (4, 5–5, 5)	0.78 (0.74–0.82)
Overall image quality	4 (4, 4–5, 5)	4 (4, 4–5, 5)	5 (4, 5–5, 5)	5 (4, 5–5, 5)	0.80 (0.76–0.85)

5-point Likert scale (1 = worst; 5 = best). Consensus ratings are reported as the median [minimum rating, interquartile range [IQR], maximum rating; Kappa: Cohen's weighted kappa (95% CIs) between two radiologists].

### 3.3 Diagnostic performance

Strong-intensity DLR demonstrated superior diagnostic concordance vs. non-DLR (*κ* = 0.908–1.00 vs. 0.882–0.963) when validated against arthroscopy-integrated radiologist assessments (Table 4). Strong-intensity DLR demonstrated perfect concordance with arthroscopy (*κ* = 1.00) vs. non-DLR (*κ* = 0.963) in patellar cartilage assessments. Strong-intensity DLR achieved perfect diagnostic concordance (*κ* = 1.00) for lateral meniscus and osseous structures, with lesion detection discrepancies ≤3% (e.g., 53/16 vs. 52/17). Muscle: Strong-intensity DLR demonstrated equivalent diagnostic accuracy vs. non-DLR (*κ* = 1.00) alongside enhanced noise reduction. Strong-intensity DLR demonstrated modest ACL/PCL concordance improvements (*κ* = 0.942/0.915 vs. 0.908/0.866) with reduced diagnostic ambiguity in ligament evaluations. Medial Meniscus: Strong-intensity DLR demonstrated enhanced diagnostic concordance vs. non-DLR (*κ* = 0.969 vs. 0.909), though subtle artifacts persisted (3/66 vs. arthroscopy 2/67). Femoral Cartilage: DLR reconstruction modes demonstrated equivalent diagnostic concordance (*κ* = 1.00) in structurally homogeneous lesions, suggesting limited incremental clinical value. Bone: Strong-intensity DLR and non-DLR demonstrated comparable diagnostic concordance (*κ* = 1.00 vs. 0.958), potentially attributable to intrinsic tissue contrast characteristics. Strong-intensity DLR enhances diagnostic reliability in noise-vulnerable anatomical zones, while requiring optimization for heterogenous tissue interfaces and artifact-susceptible regions.

**Table 4:** Diagnostic performance evaluation.

Tissue/Structure	Disease (with/without)			Arthroscopy vs. DLR (kappa value)	
	Arthroscopy	DLR = None	DLR = Strong	DLR = None	DLR = Strong
Patellar Cartilage	19/50	18/51	19/50	0.963	1
Femoral Cartilage	27/42	27/42	27/42	0.939	1
Medial Meniscus	67/2	65/4	66/3	0.909	0.969
Lateral Meniscus	53/16	52/17	53/16	0.940	1
Bone	33/36	34/35	33/36	0.958	1
ACL	67/2	66/3	67/2	0.915	0.942
PCL	57/12	56/13	57/12	0.866	0.908
Muscle	30/39	30/39	30/39	0.882	1

ACL, Anterior Cruciate Ligament; PCL, Posterior Cruciate Ligament.

## 4 Discussion

This study provides the first validation of DLR's technical superiority in 5.0T knee MRI systems, achieving simultaneous SNR enhancement while maintaining acquisition durations. DLR-reconstructed images approached arthroscopic diagnostic accuracy. Marked enhancement was demonstrated in musculature, predominantly within PDWI and T2WI sequences, with pronounced image quality improvements.

Ultrahigh-field (UHF, ≥5.0T) MRI systems, while conferring intrinsic SNR advantages, present three fundamental clinical implementation barriers: 1) Elevated thermal noise in peripheral anatomical zones; 2) Narrower-bore configurations constrain patient positioning adaptability; 3) Extended acquisition times amplify motion artifact susceptibility (24–26). This technological paradigm mandates integrating accelerated acquisitions with advanced reconstructions to harmonize diagnostic efficacy and workflow efficiency. Conventional MRI workflows operate under a fundamental trilemma paradigm: enhancing any single parameter (scan time, SNR, resolution) requires compromising at least one counterpart. Elevating signal averages (NSA) to augment SNR prolongs acquisition durations, whereas reducing spatial resolution compromises visualization of fine anatomical structures (27). Fourier reconstruction of undersampled k-space data generates Gibbs phenomenon—oscillatory artifacts originating from abrupt high-frequency truncation—obscuring tissue interfaces and lesion margins (28, 29). This study pioneers the systematic evaluation of DLR technology within 5.0T knee MRI systems to overcome these technical constraints. DLR integrates dual-domain fidelity constraints with deep feature learning to optimize spatial resolution and anatomical detail while maintaining acquisition durations. The core mechanism employs CNN-based end-to-end mapping for discriminative separation of noise and anatomic signals (30, 31). This methodology is anchored in compressed sensing theory, integrating sparse representation with nonlinear reconstruction to reconstruct high-fidelity images from sub-Nyquist k-space data (32, 33). DLR maintains acquisition efficiency while enabling enhanced visualization of anatomic structures traditionally obscured by noise (e.g., cartilage microarchitecture, meniscal fiber bundles), representing a technological breakthrough in MRI. As a “black-box” model, DLR lacks physical interpretability in its reconstruction process, which may hinder clinicians' full trust in its results. Secondly, its performance heavily depends on the training data, and it may experience performance degradation or bias when applied to different equipment, field strengths, or rare cases. Additionally, excessive denoising can lead to over-smoothing of images, potentially compromising diagnostic information from subtle textures or low-contrast lesions (34, 35).

This study systematically assessed conventional MRI sequences (T1-weighted imaging (T1WI), T2-weighted imaging (T2WI), proton density-weighted imaging (PDWI), fat-suppressed (FS)) within 5.0T knee MRI systems. Quantitative analysis demonstrated DLR significantly enhanced tissue-specific SNR. Musculature exhibited the most pronounced SNR enhancement, aligning with Jung et al.'s (36) deep residual learning framework for MRI denoising, thereby validating CNNs'adaptability in medical image reconstruction. Following the primary aim of this study, which was to evaluate the performance of the DLR algorithm against the conventional clinical standard, we did not perform formal pairwise significance tests among the three DLR intensity levels themselves (e.g., Weak vs. Medium, Medium vs. Strong). Our analysis and presentation focused on characterizing the trend and the magnitude of changes across levels descriptively (e.g., through mean/median values and graphical trends in figures), rather than on testing for statistical superiority among them. DLR demonstrates diagnostic superiority in knee pathology assessment through three cardinal advantages: DLR = Strong mode achieves complete consistency with arthroscopy (*κ* = 1.00) in structures such as patellar cartilage, lateral meniscus, and bone, with high accuracy in muscle detection (*κ* = 1.00) and effective noise reduction; for the anterior cruciate ligament (ACL) and posterior cruciate ligament (PCL), DLR = Strong slightly improves diagnostic agreement (*κ* = 0.942/0.908) compared to non-DLR modes ((*κ* = 0.915/0.866), though subtle artifacts persist in the medial meniscus (66/3 vs. arthroscopic 67/2); in homogeneous tissues like femoral cartilage, DLR matches conventional methods (*κ* = 1.00), suggesting optimization potential. Evidence demonstrates strong-intensity DLR mitigates diagnostic errors (e.g., meniscal misdiagnoses) while necessitating integration with motion-correction algorithms for precision in anatomically complex regions, supporting clinical integration to enhance diagnostic reliability.

A reduced field of view (FOV, 140 × 140 mm) was implemented to optimize spatial resolution. In obese patients demonstrating peripheral FOV signal dropout (predominantly right upper/lower quadrants) during sagittal ROI delineation, background noise quantification utilized adjacent anatomical regions' standard deviation (SD). Due to limited cross-sectional visualization efficacy for cruciate ligaments, sagittal/coronal planes were prioritized as principal analytical orientations.

This study has inherent limitations: while the sample size fulfilled minimal statistical requirements, exclusion of rare pathological entities (e.g., discoid meniscus) and pediatric cohorts constrained assessment of growth plates and related anatomical structures. The absence of disease-specific subgroup analyses precluded comprehensive evaluation of the 5.0T reconstruction algorithm's pathological generalizability. Diagnostic validation was limited to comparative analysis between DLR's “None” and “Strong” modes, excluding intermediate/weak intensity parameters—constraining conclusion generalizability; the protocol's restriction to 2D acquisitions left 3D sequence applicability unvalidated. Data were exclusively acquired from United Imaging 5.0T systems, lacking cross-platform validation across multivendor/multifield-strength systems. Future multicenter investigations are warranted to improve technical generalizability.

## 5 Conclusion

This study establishes the clinical efficacy of deep learning reconstruction (DLR) in 5.0T knee MRI through significant SNR enhancement (12.61%–350.63%), arthroscopy-validated diagnostic superiority (*κ* = 0.908–1), providing a methodological foundation for multi-anatomical applications of ultrahigh-field MRI and optimized workflow efficiency.

## Data Availability

The original contributions presented in the study are included in the article/Supplementary Material, further inquiries can be directed to the corresponding author.
